# Lactate provides a strong pH-independent ventilatory signal in the facultative air-breathing teleost *Pangasianodon hypophthalmus*

**DOI:** 10.1038/s41598-017-06745-4

**Published:** 2017-07-25

**Authors:** Mikkel T. Thomsen, Tobias Wang, William K. Milsom, Mark Bayley

**Affiliations:** 1 Department of Bioscience, Zoophysiology, Aarhus University, Aarhus, Denmark; 2 Department of Zoology, University of British Columbia, Vancouver, BC Canada

## Abstract

Fish regulate ventilation primarily by sensing O_2_-levels in the water and arterial blood. It is well established that this sensory process involves several steps, but the underlying mechanisms remain frustratingly elusive. Here we examine the effect of increasing lactate ions at constant pH on ventilation in a teleost; specifically the facultative air-breathing catfish *Pangasianodon hypophthalmus*. At lactate levels within the physiological range obtained by Na-Lactate injections (3.5 ± 0.8 to 10.9 ± 0.7 mmol L^−1^), gill ventilation increased in a dose-dependent manner to levels comparable to those elicited by NaCN injections (2.0 µmol kg^−1^), which induces a hypoxic response and higher than those observed in any level of ambient hypoxia (lowest PO_2_ = 20 mmHg). High lactate concentrations also stimulated air-breathing. Denervation of the first gill arch reduced the ventilatory response to lactate suggesting that part of the sensory mechanism for lactate is located at the first gill arch. However, since a residual response remained after this denervation, the other gill arches or extrabranchial locations must also be important for lactate sensing. We propose that lactate plays a role as a signalling molecule in the hypoxic ventilatory response in fish.

## Introduction

Animals rely on a suite of physiological adaptations to maintain sufficient supply of O_2_ to the respiring tissues when exposed to hypoxia. These adaptations vary amongst vertebrate classes, but normally include increased ventilation and cardiovascular adjustments, with the exact type and magnitude of the response being affected by the severity and duration of the hypoxia^1–3^. The first step required in a hypoxic response is obviously a sensing mechanism responding to changes in O_2_ levels. Yet, despite much effort, the mechanisms underlying O_2_ sensing remain frustratingly elusive, even in the extensively studied mammalian carotid body^4^. Determining the modality of O_2_ sensing is paramount in understanding ventilatory regulation and hypoxic responses, which are typically assessed by hypoxia exposure *per se* or by injection of various chemicals such as NaCN, which stimulates oxygen sensitive receptors^5, 6^.

The capacitance of O_2_ is around 25–30 times lower in water than in air. Hence, water-breathing animals must ventilate more than air-breathers to meet their metabolic O_2_ demand^7, 8^. These high ventilation rates result in a low partial pressure of CO_2_ in arterial blood, and a ventilatory regulation of the arterial CO_2_-level would unavoidably compromise O_2_ uptake. Hence, pH-regulation is mainly achieved by transepithelial H^+^/HCO_3_^–^exchange in fish^9, 10^. In mammals and other air-breathing tetrapods, the ventilatory requirement for O_2_ uptake is lower, leading to higher arterial PCO_2_ levels^7, 8^. This increase in combination with the high O_2_ availability has changed the mode of ventilatory regulation in air breathers into being primarily controlled by H^+^/CO_2_ changes under normoxic conditions^11^. Thus the oxygen ventilatory signal in most mammals has utility only in situations outside the normal adaptive range such as at high altitude, diving, burrowing or during pulmonary illness, but is the dominant regulatory mechanism of ventilation in fish.

Hardarson *et al*.^12^ showed for the first time in a vertebrate that the lactate ion *per se* gives a stimulation of ventilation in rats that is separate from the effect of the normally accompanying acidosis^12, 13^ Chang *et al*.^14^ recently documented the *olfactory receptor 78* to be involved in this response by describing a high lactate affinity of this receptor in the carotid body. By knocking out the coding gene (*Olfr78*, also known as *Or51e2*) in mice they abolished the carotid body responses to both lactate ions and hypoxia^14^. Thus, it seems that lactate ions induce a ventilatory response similar to hypoxemia (low blood O_2_ levels) and the authors argued that lactate and lactate sensing may have an important role in enhancing the hypoxic ventilatory response. Lactate is produced by most vertebrate cells under hypoxic conditions due to a build-up of the glycolytic end-product pyruvate, which is enzymatically converted to lactate and released to the blood. Since lactate is produced by all vertebrates, this lactate-induced component of the hypoxic response may be a universal trait amongst vertebrates, making it timely to study the influence of the lactate ion on ventilation in other vertebrate classes.

In this study we explore the presence of a pH-independent lactate ventilatory response in a teleost, namely the facultative air-breathing fish *Pangasianodon hypophthalmus*, which uses gills and a modified swim bladder for O_2_ uptake. Since the teleost first gill arch is ontogenetically homologous to the mammalian carotid body^15, 16^, we also examined the lactate responses following denervation of the first gill arch. We hypothesised that this denervation should reduce or abolish the lactate ventilatory response. Finally, we examined the cardiorespiratory responses to progressive hypoxia and/or hypercarbia to simulate natural conditions^17–20^ for comparison of the response elicited by lactate ions.

## Results

### Intra-arterial injections

**Figure 1:**
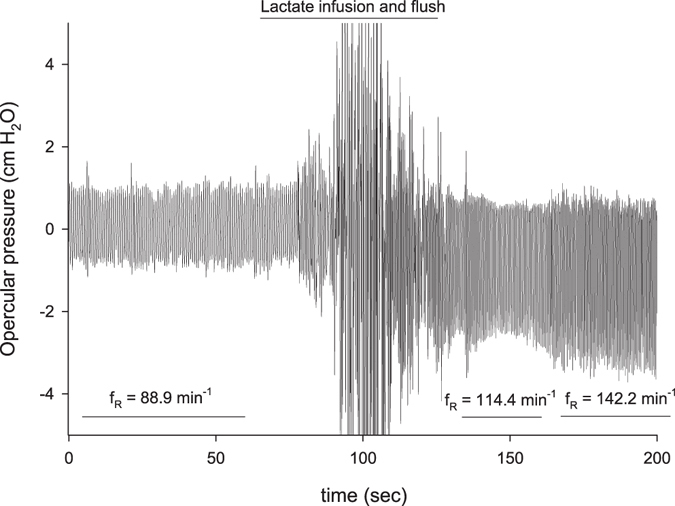
A representative trace of the opercular pressure data around and the time of a lactate injection. This trace is an example of a 1 M lactate injection dose in a non-denervated subject. Average f_R_ is shown for three intervals; before injection, just after the injection, and when it reaches peak level.

**Figure 2:**
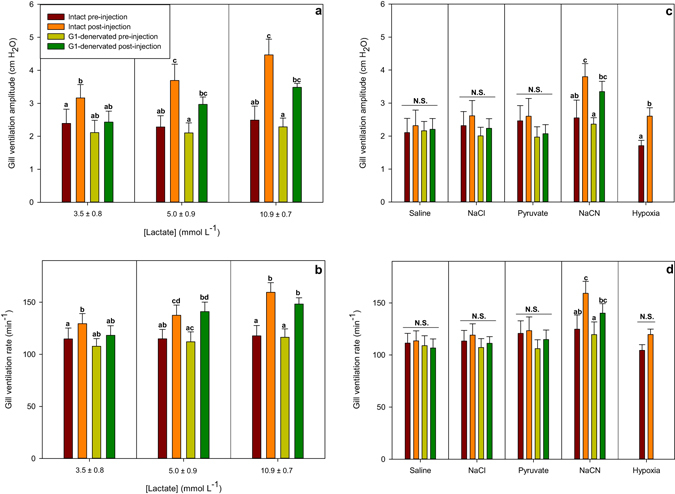
The effect of the injections and hypoxia on gill ventilation. (**a**) Pre-injection and post-injection gill ventilation amplitude at the three lactate levels for intact and G1-denervated fish. (**b**) Pre-injection and post-injection gill ventilation rate at the three lactate levels for intact and G1-denervated fish. (**c**) Pre-injection and post-injection gill ventilation amplitude following the control injections for intact and G1-denervated fish as well as the maximal response to progressive ambient hypoxia (normoxic pre-exposure and maximum hypoxic response averaged between all subjects are shown) reached at P_w_O_2_ = 47.5 ± 2.5 mmHg. (**d**) Pre-injection and post-injection gill ventilation rate following the control injections for intact and G1-denervated fish as well as the maximal response to progressive ambient hypoxia (normoxic pre-exposure and maximum hypoxic response are shown) reached at P_w_O_2_ = 47.5 ± 2.5 mmHg. A mixed model ANOVA followed by a pairwise comparison between each point for the different injections was used to analyse the injection data. In hypoxia, a two-tailed paired T-test was used. Bars sharing a letter within a treatment are not significantly different from each other. Results are shown as mean + s.e.m. For G1 and hypoxia n = 6. For Intact n = 8.

**Figure 3:**
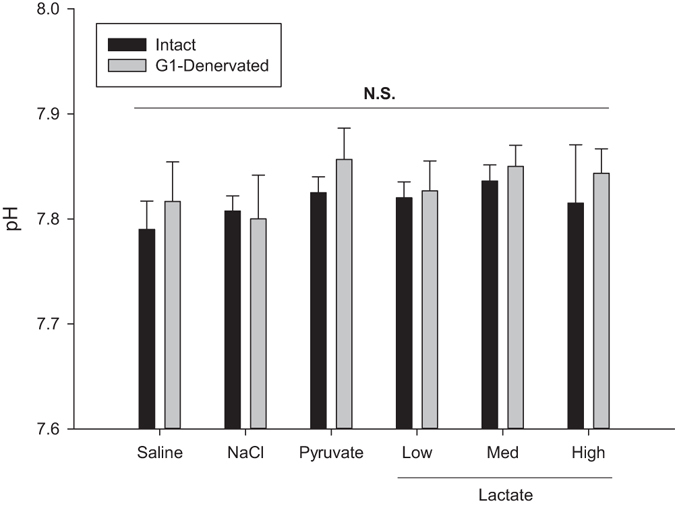
The effect on arterial pH following injections. Bar plot showing the arterial pH measured 5 min after injection of the given substance. Low, Med and High refer to the three doses of lactate injected where the measured lactate concentrations were 3.5 ± 0.8, 5.0 ± 0.9, and 10.9 ± 0.7 mmol L^−1^, respectively. No significant differences between treatments or between intact and G1-denervated fish were found using a mixed model ANOVA. Results are shown as mean + s.e.m. For G1, n = 6. For Intact n = 8.

Intra-arterial injections of lactate in the non-denervated fish caused a dose-dependent increase in both gill ventilation rate (f_R_, p = 0.0042, p = 0.0003, p = 0.0003 for the low, medium and high lactate doses, respectively; mixed model ANOVA with pairwise comparison and Benjamini-Hochberg p-value adjustment, n = 14, Figs 1 and 2a) and gill ventilation amplitude (V_amp_, p = 0.006, p = 0.0003, p = 0.0002 for the low, medium and high lactate doses, respectively, mixed model ANOVA with pairwise comparison and Benjamini-Hochberg p-value adjustment, n = 14, Figs 1 and 2b) independent of changes in pH (pH = 7.82 ± 0.01, no difference between treatments, p = 0.70, or between G1-denervated and non-denervated fish, p = 0.37, mixed model ANOVA, n = 14, Fig. 3). This response also included air-breathing at the two higher doses (25% and 50% non-denervated fish and 0% and 33% G1-denervated fish performed at least 1 observable air-breath following the medium and high lactate doses, respectively), but this response could not be quantified further in our experimental setup. While denervation of the first gill arch failed to completely abolish the lactate ventilatory response, it did cause a reduction. The response of the G1-denervated fish to elevated lactate was still significant at the two highest doses (Fig. 2; f_R_ p = 0.10, p = 0.003 and p = 0.003 at the low, medium and high lactate doses, respectively; V_amp_ p = 0.27, p = 0.003 and p = 0.002, at the low, medium and high lactate doses, respectively, mixed model ANOVA with pairwise comparison and Benjamini-Hochberg p-value adjustment, n = 14). None of the control injections – isosmotic saline, 1 M NaCl, or pyruvate – affected ventilation (no air-breaths were observed and no increase in either f_R_ or V_amp_) or pH (Figs 2 and 3), confirming that the responses to other treatments were not a result of stress from injections, increased blood pressure, altered osmolality/[Na^+^], or a metabolic response from the conversion of lactate to pyruvate.

NaCN injections (2.0 µmol kg^−1^) initiated clear ventilatory responses in both intact and denervated fish (Fig. 2c,d) in addition to air-breathing (50% of the fish in both groups performed at least 1 observable air-breath) and escape behaviour (not quantified). The time between injection and a visible ventilatory response was similar for lactate and NaCN injections, with the peak response occurring after 54 ± 4 sec for lactate injections and after 46 ± 10 sec for NaCN injections.

### Hypoxia and hypercarbia

**Figure 4:**
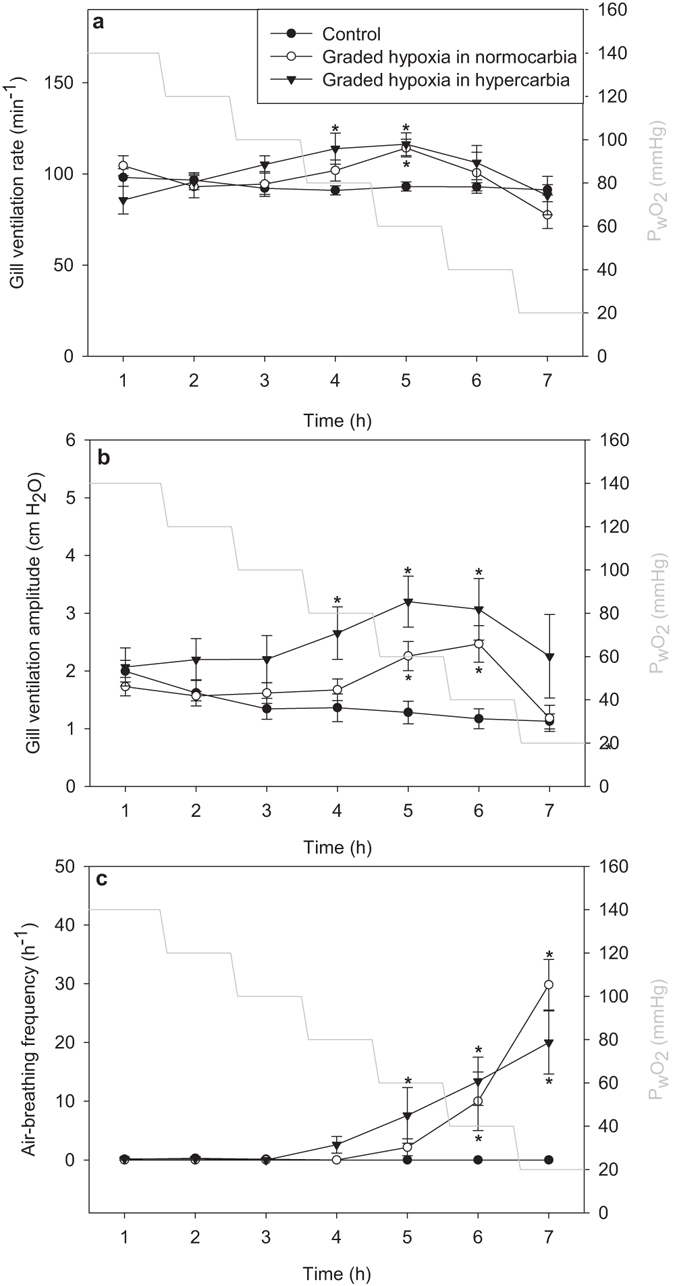
The effect of progressive hypoxia on ventilation. (**a**) The effect of progressive hypoxia on gill ventilation rate. (**b**) The effect of progressive hypoxia on gill ventilation amplitude. (**c**) The effect of progressive hypoxia on air-breathing frequency. The control group were kept in normoxic, normocarbic water throughout the measurements. The group with constant hypercarbia were exposed to 22.5 mmHg PCO_2_ for 1–2 hours before adjusting water PO_2_. Results are shown as mean ± s.e.m. For control n = 10, for the other groups n = 6.

**Figure 5:**
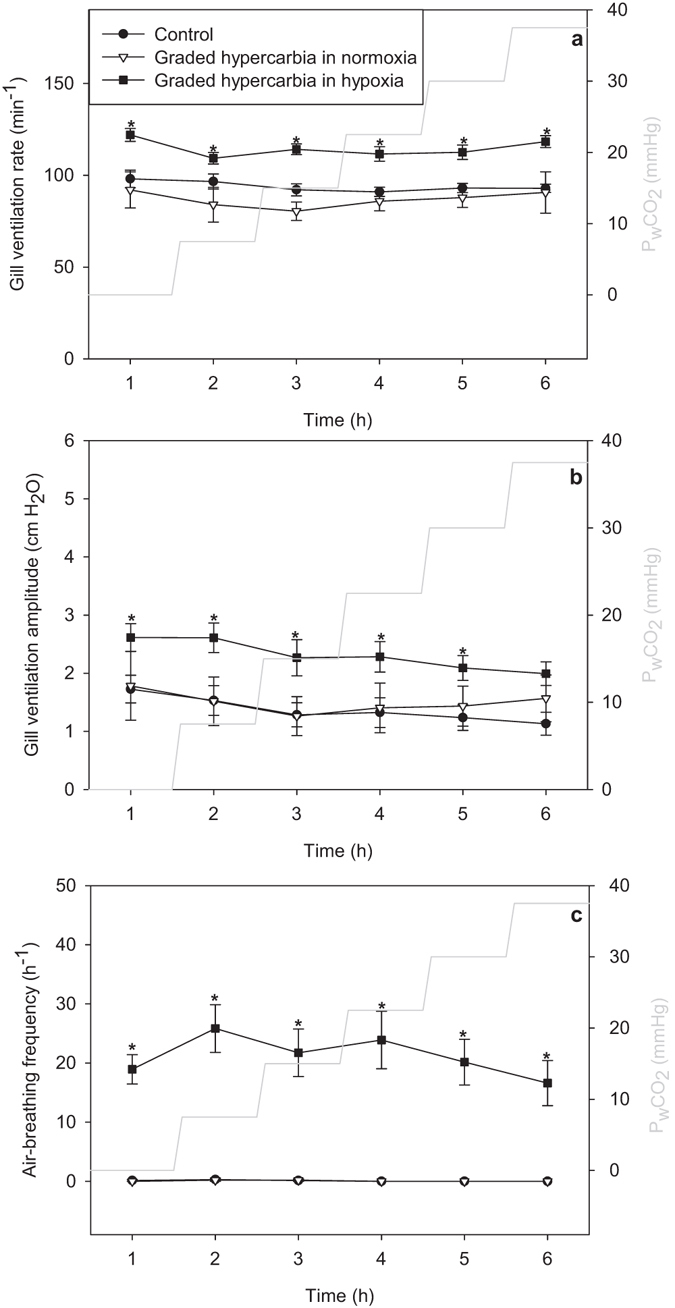
The effect of progressive hypercarbia on ventilation. (**a**) The effect of progressive hypercarbia on gill ventilation rate. (**b**) The effect of progressive hypercarbia on gill ventilation amplitude. (**c**) The effect of progressive hypercarbia on air-breathing frequency. The control group were kept in normoxic, normocarbic water throughout the measurements. The group with constant hypoxia were exposed to 40 mmHg PO_2_ for 1–2 hours before adjusting water PCO_2_. Results are shown as mean ± s.e.m. For control n = 10, for the other groups n = 6.

Hypoxia induced changes in gill ventilation (f_R_ p = 0.002; V_amp_ p = 0.002, mixed model ANOVA, n = 22), with moderate decreases in water PO_2_ (P_w_O_2_ = 40–60 mmHg) eliciting increased gill ventilation with the largest elevation in V_amp_, whereas more severe hypoxia (P_w_O_2_ < 40 mmHg) returned gill ventilation close to pre-experimental levels (Fig. 4a,b). The reduction in gill ventilation corresponded with an increased air-breathing frequency (Fig. 4c). The same pattern was observed with progressive hypoxia in water with a constant elevated water PCO_2_ (P_w_CO_2 = _22.5 mmHg) with a slight tendency for a further rise in gill ventilation compared to progressive hypoxia in normocarbic water (Fig. 4, not significant at any level of P_w_O_2_ for either f_R_ or V_amp_, mixed model ANOVA with pairwise comparison, n = 22). The maximum response to hypoxia was reached at 47.5 ± 2.5 mmHg (mean ± s.e.m, Fig. 2c,d). Progressive hypercarbia in normoxia failed to elicit an effect on ventilation (Fig. 5, f_R_ p = 0.28; V_amp_ p = 0.81, mixed model ANOVA, n = 22). With hypoxia added to the progressive hypercarbia, the ventilation was increased (Fig. 5, f_R_ p = 0.0002, V_amp_ p = 0.01, mixed model ANOVA, n = 22). However, despite this elevation, the progression of the curve is similar to that of the control group (Fig. 5a, p = 0.78, mixed model ANOVA, n = 22). Virtually no air-breathing was observed with progressive hypercarbia in isolation of hypoxia, while with constant hypoxia present the air-breathing frequency was increased to 23.3 ± 1.6 h^-1^. The heart rate response to hypoxia and hypercarbia is shown in supplementary Fig. S1.

## Discussion

This study reveals a substantial ventilatory response to increased circulating lactate ions at physiologically relevant concentrations, independent of changes in plasma pH. This response to lactate in non-denervated fish was strikingly similar to that stimulated by NaCN (Fig. 2), a potent inducer of hypoxic responses^5^ and similar, but larger than that caused by moderate aquatic hypoxia (Figs 2 and 4). These similarities, in combination with the recent discovery that lactate ions mediated the hypoxic ventilatory response in mammals^14^, suggest that a similar mechanism is present in *P. hypophthalmus* when lactate levels increase.

The failure to completely remove the ventilatory response by denervation of the first gill arch (Fig. 2) indicates that the putative lactate receptors are not limited to the first gill arch, whose sensory cells are considered homologous to the carotid body glomus cells^15, 16^, central in initiating the response in mammals^14^. Denervation of the first gill arch had a similar effect on the ventilatory response to NaCN injections, indicating that the hypoxic response in this species is not isolated to the first gill arch. This similarity between the effect of denervation on the responses following lactate and NaCN is consistent with our interpretation that the lactate ventilatory response is linked to the general O_2_ sensing pathway (Fig. 2). Given the resemblance in response between the two chemical stimuli and given the extensive evidence for O_2_ sensing in fish being performed by neuroepithelial cells (NECs)^15, 21, 22^, it is likely that the lactate response is mediated by a NEC type, although our data cannot determine whether the two stimuli mediate their response through the same NEC type. Several NEC types have been identified that respond to different stimuli or groups of stimuli, including at least O_2_, CO_2_, H^+^, NH_4_^+^, CO, NO and H_2_S^23–29^, and are therefore typically divided into subpopulations^25, 30, 31^. These subpopulations of NECs differ in distribution and there seems to be considerable interspecific variation in the distribution of NEC cell types, although understanding of subpopulation distribution patterns between species is still very limited^21, 25, 32, 33^. Identifying the location of the putative lactate receptors e.g. by immunolabeling or measuring the response of lactate on isolated NECs by patch-clamping techniques would be an interesting continuation of the present study.

It might be argued that the lactate response in the present study was a secondary metabolic response because increased lactate concentrations might elevate O_2_ consumption^34, 35^. However, the rapid ventilation changes and the magnitude of the changes following lactate injections render this possibility unlikely. Ventilatory increases began shortly after the injection (within 30 sec) and the peak occurred after 53 ± 4 sec (mean ± s.e.m.) after lactate and NaCN injections with some inter- and intra-individual variation and no clear correlation between either magnitude of the response, injected substance or denervated/non-denervated state. This time lag is likely due to the transit time from point of injection (dorsal aorta) to the gills. Furthermore, had lactate oxidation increased metabolic rate and hence provided a secondary stimulation of ventilation, we would have expected a similar stimulation following pyruvate injections, which was not the case (Fig. 2).

*P. hypophthalmus* meets its metabolic O_2_ demand almost exclusively from gill ventilation in normoxic water, but resorts to air-breathing in hypoxic water below the critical oxygen tension (i.e. below the O_2_ tension where the standard metabolic rate cannot be maintained solely through branchial gas exchange – around 60 mmHg at 27 °C)^36^. Below the critical oxygen tension it is still able to uphold its aerobic metabolism completely by air-breathing and does not produce lactate above the detection limit (ref. 36 and Damsgaard et al., *submitted*). With this in mind, it is not surprising that at ambient O_2_ levels above the critical oxygen tension, the response to reduced P_w_O_2_ is similar to purely water-breathing fish. Thus, at PO_2_ tensions above 60 mmHg we saw an increased gill ventilation, stemming mainly from increased V_amp_, as was the case with lactate and NaCN injections (Figs 2 and 4)^10, 21, 37^. Hypoxia impairs growth in this species^38, 39^, in part from elevated energetic costs of air-breathing^40^, but the present data suggest that increased gill ventilation in moderate hypoxia might also contribute to the increased energetic costs. In more severe hypoxia (P_w_O_2_ < 40 mmHg), gill ventilation started to decline as air-breathing rose (Fig. 4), which is a pattern similar to several other air-breathing fish^41–46^. This reduced gill ventilation, is argued to alleviate branchial loss of the O_2_ taken up by air-breathing^47^, and is in agreement with Lefevre *et al*.^36^, where no indication of branchial O_2_ loss was observed at a P_w_O_2_ of 2kPa (=15 mmHg)^36^. The increased ventilation from ambient hypoxia was of a lower magnitude than that observed with high doses of lactate and the NaCN injections (2.0 µmol kg^−1^). The main part of this difference is probably due to the injections giving short, peak responses, whereas changes in water PO_2_ cause the ventilation to stabilise at a new level. Changing the water PO_2_ took several minutes whereas the injections elicit sudden changes which might trigger a stronger hypoxic response, including the observed escape behaviour, which is a common reaction to acute hypoxia, particularly in active fishes such as *P. hypophthalmus* ^2, 48, 49^. This escape behaviour probably potentiates the ventilatory increase as the activity level becomes higher than at any level of progressive hypoxia. Escape behaviour was rare in the hypoxia treatment, likely due to the step-wise P_w_O_2_ adjustment, making the changes less acute.

Previous studies in *P. hypophthalmus* have shown that blood pH equilibrates rapidly when the PCO_2_ of the ambient water changes^20, 50^. In water with a PCO_2_ of 34 mmHg, blood pH fell rapidly from 7.8 to 7.3 at 27 °C. In this study we observed no ventilatory effect of hypercarbia up to 37.5 mmHg, the maximum level tested in this study and probably in the high range that these fish might experience naturally^20^. Thus, blood pH changes within a physiologically relevant range are unlikely to have a significant impact on ventilation in this species. It is possible, however, that a ventilatory CO_2_/H^+^ response might appear at higher P_w_CO_2_ levels than used in the present study, as some species only show a response at very high P_w_CO_2_, where the physiological importance under natural conditions is minor.

These results clearly show that increases in circulating lactate ions in a concentration range of physiological relevance, affect ventilation in fish. However, as the hypoxic ventilatory response usually occurs very rapidly after experiencing hypoxia^2, 3^, lactate would need to be produced locally near lactate/hypoxia sensing cells if of importance in triggering the hypoxic ventilatory response. This is hypothesised to be the case in mammalian glomus cells due to the markedly reduced hypoxic ventilatory response in *Olfr78*-knockout mice^14^. These authors argue that due to the high metabolic rate of these cells, they produce lactate even after minor reductions in circulating O_2_. With the present data we cannot rule out that the mechanism in teleosts might differ from that suggested in mammals: That the main function of the lactate ventilatory response might be to enhance ventilation as circulating lactate starts to increase (e.g. during intensive swimming) rather than to function from locally produced lactate. It is tempting to speculate on this lactate role as plasma lactate levels in *P. hypophthalmus* immediately after intense swimming are usually in the range 7–10 mmol L^−1^ (unpublished data), corresponding well with the highest lactate levels reached in this study.

The finding that lactate ions dramatically stimulate ventilation in fish opens an array of new questions for future studies: How general is the hypoxic lactate response amongst vertebrates? Is *Olfr78* also responsible for the lactate sensing in fish, or is an alternative mechanism involved? Is locally produced lactate important in inducing a hypoxic response as has been suggested in mice^14^, or is the response mainly elicited from circulating lactate levels as tested in this study? Answers to these kinds of questions will provide significant input in determining the role of lactate as a hypoxic signalling molecule, and enhance the understanding of the complex O_2_ sensing mechanism.

## Methods

### Experimental animals

Juvenile *Pangasianodon hypophthalmus* (Sauvage, 1878) were obtained from Credo Fish (Denmark) and kept at a recirculation system at Aarhus University at 33 °C for several months until experimentation at a 12 h:12 h light-dark cycle. The water was maintained normoxic and P_w_O_2_ was monitored continuously. The fish were kept in the same circulating system (same water), but were randomly divided into two 1000 L tanks on arrival to Aarhus University. The animals were fed trout pellets to satiation every day and appeared healthy.

### Animal preparation

The animals were anaesthetised to a surgical level in an aqueous solution of benzocaine (150 mg L^−1^), and moved to a surgical setup with irrigation of the gills with aerated benzocaine solution (75 mg L^−1^). A PE50 catheter was inserted into the dorsal aorta, extended through a hole in the rostrum and secured with a cuff ^51^. Another PE50 catheter was inserted into the opercular cavity through the operculum and secured. In testing the effects of changing ambient gas levels no further surgery was performed (n = 34, mass = 677 ± 73 g). The fish receiving injections (n = 14, mass = 467 ± 32 g) were subdivided into an intact group (n = 6; no further surgery), a G1-denervated group (n = 6; ablation of the IX^th^ and X^th^ cranial nerves branches to the first gill arch through a small incision through the membrane in the opercular cavity) and a sham group (n = 2; the same nerves were exposed, but not severed). Post operation, all fish were allowed at least 24 h to recover in well-aerated water at 33 °C. Denervations were confirmed *post mortem*. All procedures were conducted according to the guidelines of the Danish Law on Animal Experiments and were approved by the Danish Ministry of Food, Agriculture and Fisheries (2016-15-0201-00865).

### Intra-arterial injections

Following recovery in a swim tunnel at 0.5 body lengths sec^−1^ (the same speed was kept through the experiment), the opercular catheter was connected to a pressure transducer (PX600, Irvine, CA, USA) through a swivel system (Instech Laboratories, Inc., Plymouth Meeting, PA, USA) and the following substances were injected through the dorsal aorta catheter: isosmotic saline, to identify effects related to the act of injection or increase blood volume; 1 M NaCl (Merck, Kenilworth, NJ, USA), to identify effects of increased osmolarity or increased [Na^+^]; 0.1 M Na-pyruvate (Sigma-Aldrich Denmark A/S, Copenhagen, Denmark), to identify effects of pyruvate; 0.3 M Na L-lactate (Sigma-Aldrich), 0.6 M Na L-lactate, 1 M Na L-lactate, all of which were to identify if a dose dependent lactate ion response was present; 2.0 µmol kg^−1^ NaCN (Merck), for determining the transit time to the O_2_ sensing cells, and for chemically inducing a hypoxic response. Injection volumes were 1 mL for control injections (isosmotic saline, 1 M NaCl, and pyruvate). Na L-lactate, Na-pyruvate and NaCN were dissolved in isosmotic saline. For Na L-lactate, the volumes were adjusted by giving all fish a similar mass specific dose (1 ml kg^−1^) of Na L-lactate (1^st^ dose) and measuring [lactate]. Subsequent injection volumes were adjusted according to this measurement to attain similar [lactate] in all fish for easier comparison. Injections never exceeded 1 ml. The lactate dosages were chosen after a pilot study aimed at reaching physiological relevant lactate levels, and the NaCN concentration based on a pilot study aimed at triggering the fish to air-breathe most of the time. The other injections were based on the lactate dosages to eliminated alternative causes of the response. After each injection, catheters were flushed with saline. Injections were staggered by at least 30 min and ventilation always returned to around pre-injection levels before continuing. The order of the control injections were randomized, but the lactate injections were always given last, to avoid an effect of lactate if levels had not returned to normal before the next injection. Ventilation was monitored for 5 min post-injection, and then 150 µL blood was withdrawn, and plasma pH (SevenCompact pH-meter, Mettler-Toledo Ltd., Greifensee, Switzerland) and blood [lactate] (Accutrend Plus, Roche Diagnostics Limited, Rotkreuz, Switzerland) were measured^52^.

The responses of sham-operated fish, with exposed but un-severed branchial nerves, were indistinguishable from those of control fish for any injected substances and data from both groups were thus combined to a single control group (n = 8) for all figures and further data analysis.

### Effect of ambient gas levels

Following recovery, a bottomless Plexiglas pyramid with an air in- and outflow at the top was lowered into the recovery tank where water level was regulated in such way that an air space sufficiently large for air-breathing was present in the top. The two catheters were connected to pressure transducers (PX600, Irvine, CA, USA) to measure heart rate (f_H_), gill ventilation rate (f_R_), and amplitude (V_amp_), and the fish left for 1–2 hours to recover after covering the setup with dark plastic. A respirometer system (Respirometer, v. 1.5.0.c Aarhus University, Denmark) coupled with optical O_2_ probes (VisiFerm DO, Hamilton Process Analytics, NV, USA) regulated temperature and P_w_O_2_^36^, while P_w_CO_2_ was regulated using an Oxyguard Pacific system coupled with a G10ps CO_2_ probe (Oxyguard International A/S, Farum, Denmark). For measurements of air-breathing frequency, a constant flow of normoxic, normocarbic air was sent through the air-space and exhaust gas run through an O_2_ and CO_2_ gas analyser (Servomex 5200 multipurpose – Servomex, Crowborough, UK). The fish were then divided into 5 groups and subjected to one of the following treatments: A) Progressive hypoxia (n = 6, P_w_O_2_: > 140–20 mmHg, stepwise over 7 hours), B) Progressive hypercarbia (n = 6, P_w_CO_2_: 0–37.5 mmHg, stepwise over 6 hours, C) Progressive hypoxia in constant hypercarbia (n = 6, P_w_CO_2_: 22.5 mmHg, P_w_O_2_ as in A), D) Progressive hypercarbia in constant hypoxia (n = 6, P_w_O_2_: 40 mmHg, P_w_CO_2_ as in B), E) control (n = 10, P_w_O_2_: > 140 mmHg, P_w_CO_2_: 0 mmHg for 7 hours). Each exposure step lasted 1 hour to allow ventilation to stabilise. 1 h steps were chosen as changing the water PO_2_ and PCO_2_ took several minutes, preventing the recording of reliable acute hypoxia responses directly comparable to those induced by the chemical injections.

### Data sampling and analysis

Measurements from the pressure transducers and the gas analyser were collected with a MP100 BIOPAC system (Biopac Systems Inc., Santa Barbara, CA, USA) at 200 Hz and stored in AcqKnowledge v. 3.9.1, where data was smoothed by a factor 25 to reduce the noise and allow for automatization of the analysis in most data. In the ambient gas experiment the reported values are taken from the last 15 min of each hour of the progressive hypoxia or hypercarbia (45 minutes to stabilise). Spontaneous high activity levels were excluded both to reduce the random variation and because of the high level of noise on the signals during these periods. In the intra-arterial injections experiment, the reported post-injection values are the peak response averaged over 30 seconds whereas the pre-injection values are the average of 30 second immediately prior to injection. Periods with noise due to movement were few and were avoided in the analysis. All data is available at https://pastebin.com/44d7jRdG.

### Statistics

All data are presented as mean ± s.e.m. A two-way mixed model analysis of variance followed by a pairwise comparison and a Benjamini-Hochberg p-value correction was used to analyse most results. For the injection experiment, subject was included as a random factor and treatment and group as fixed factors. The p-values reported for an effect of an injection are the adjusted p-values from the pairwise comparison between pre- and post-injection for a given substance for a given variable. For the ambient gas experiment, subject was included as a random factor and time and group included as fixed factors. Pairwise comparison was made at each time-point, comparing the specific treatment to the control group. The analysis was performed using InVivoStat software (v. 3.6.0.0). All data were tested for variance homoscedacity with residual plots and normality with a normal probability plot. In the few cases of heteroscedacity (air-breathing data and gill ventilation amplitude data for hypoxia exposure in normocarbia) the data was rank-transformed prior to performing the mixed model ANOVA. The maximal effect of hypoxia on gill ventilation variables (Fig. 2c,d.) were analysed with a two tailed paired T-test. Differences were considered significant when p < 0.05.

## Electronic supplementary material

Supplementary Information
