# Supplementary material for: Lactate provides a strong pH-independent ventilatory signal in the facultative air-breathing teleost Pangasianodon hypophthalmus
Source: Sci Rep. 2017 Jul 25;7:6378. doi: 10.1038/s41598-017-06745-4 (PMC5527003; doi:10.1038/s41598-017-06745-4)
Supplement: Supplementary file 1 — Supplementary Information [file 41598_2017_6745_MOESM1_ESM.pdf]

Lactate provides a strong pH-independent ventilatory signal in the facultative air-breathing teleost *Pangasianodon hypophthalmus*

Mikkel Thy Thomsen, Tobias Wang, William K. Milsom and Mark Bayley

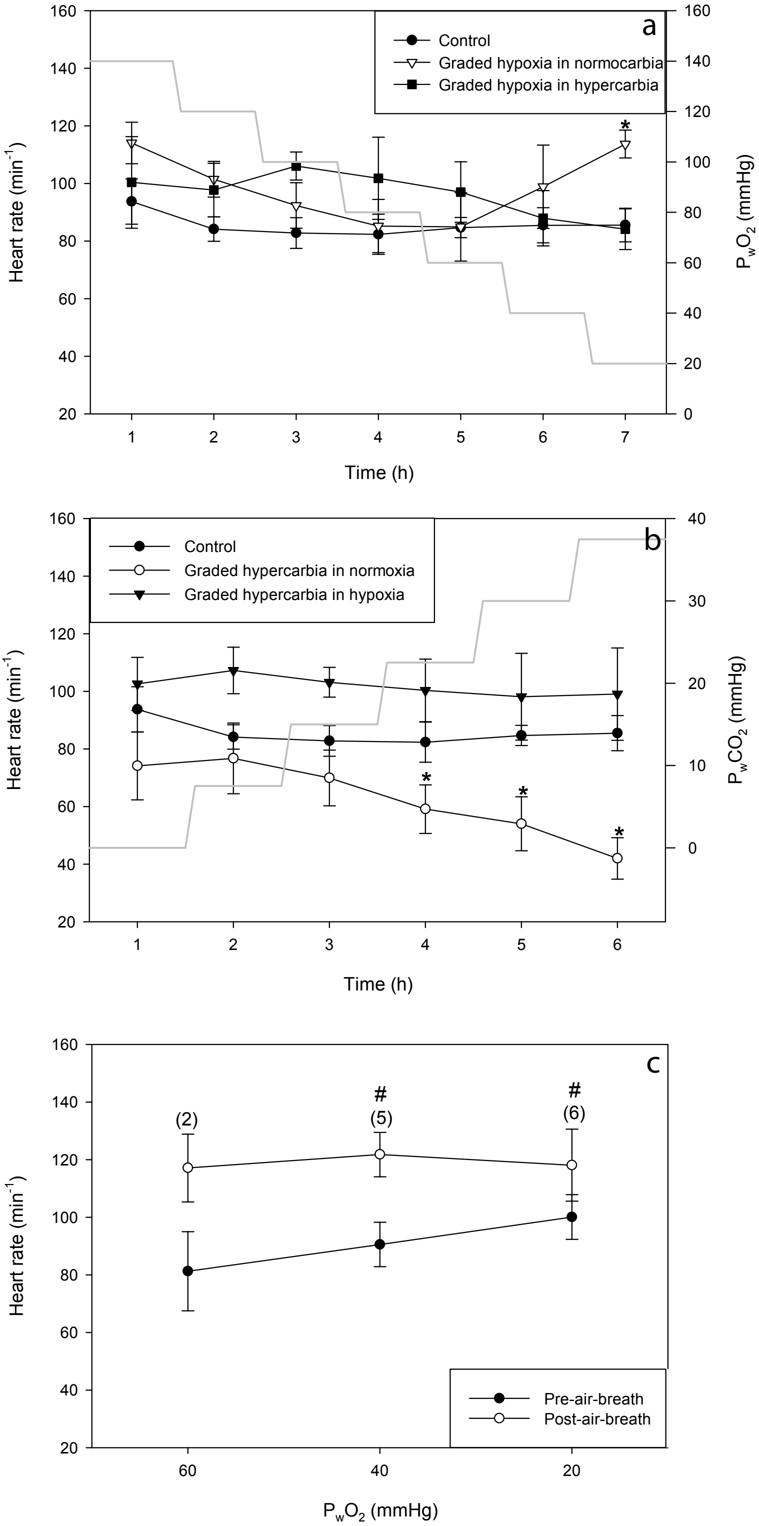

Supplementary figure S1: The effect of ambient gas levels on heart rate. a) The effect of progressive hypoxia on heart rate. The P_wCO_2 in the group exposed to hypercarbia was 22.5 mmHg. b) The effect of progressive hypercarbia on heart rate. The P_wO_2 in the group exposed to hypoxia was 40 mmHg. For both a) and b) The control group was kept in normoxia and normocarbia throughout the measurement. Asterisks indicate a significant difference from the control group, determined by a mixed model ANOVA followed by a pariwise comparison. c) The heart rate prior to and immidiately after an air-breath (average of 10 beats). At low P_wO_2 the air-breathing frequency is high, and the heart rate does not return to normoxic levels before the next air-breath, explaning wh the average heart rate in progressive hypoxia (a) increases at low P_wO_2. The numbers in c) indicates number of fish that air-breathed at the given PwO2 (total n = 6). # indicate a significant difference between pre- and post-air-breathing, determined by a two tailed paired T-test followed by Benjamini-Hochberg p-value adjustment.
